# An Overview of the Experimental Studies on the Electrical Conductivity of Major Minerals in the Upper Mantle and Transition Zone

**DOI:** 10.3390/ma13020408

**Published:** 2020-01-15

**Authors:** Lidong Dai, Haiying Hu, Jianjun Jiang, Wenqing Sun, Heping Li, Mengqi Wang, Filippos Vallianatos, Vassilios Saltas

**Affiliations:** 1 Key Laboratory of High-Temperature and High-Pressure Study of the Earth’s Interior, Institute of Geochemistry, Chinese Academy of Sciences, Guiyang 550002, China; dailidong@vip.gyig.ac.cn (L.D.); jiangjianjun@vip.gyig.ac.cn (J.J.); sunwenqing@vip.gyig.ac.cn (W.S.); liheping@vip.gyig.ac.cn (H.L.); wangmengqi@vip.gyig.ac.cn (M.W.); 2 UNESCO Chair in Solid Earth Physics and Geohazards Risk Reduction, Hellenic Mediterranean University, 3 Romanou St., Chalepa, GR73 133 Chania, Greece; fvallian@geol.uoa.gr; 3 Department of Geophysics–Geothermics, Faculty of Geology and Geoenvironment, National and Kapodistrian University of Athens, 15772 Athens, Greece

**Keywords:** electrical conductivity, impedance spectroscopy, mantle, olivine, pyroxene, garnet, wadsleyite, ringwoodite, high-pressure, high-temperature

## Abstract

In this paper, we present the recent progress in the experimental studies of the electrical conductivity of dominant nominally anhydrous minerals in the upper mantle and mantle transition zone of Earth, namely, olivine, pyroxene, garnet, wadsleyite and ringwoodite. The main influence factors, such as temperature, pressure, water content, oxygen fugacity, and anisotropy are discussed in detail. The dominant conduction mechanisms of Fe-bearing silicate minerals involve the iron-related small polaron with a relatively large activation enthalpy and the hydrogen-related defect with lower activation enthalpy. Specifically, we mainly focus on the variation of oxygen fugacity on the electrical conductivity of anhydrous and hydrous mantle minerals, which exhibit clearly different charge transport processes. In representative temperature and pressure environments, the hydrogen of nominally anhydrous minerals can tremendously enhance the electrical conductivity of the upper mantle and transition zone, and the influence of trace structural water (or hydrogen) is substantial. In combination with the geophysical data of magnetotelluric surveys, the laboratory-based electrical conductivity measurements can provide significant constraints to the water distribution in Earth’s interior.

## 1. Introduction

Results in the geophysical field observations from magnetotelluric (MT) and geomagnetic deep sounding (GDS) surveys have already confirmed the existence of anomalous high electrical conductivity (EC) zones (conductivities: 10^−1^–10^−2^ S/m), in the global and regional geotectonic units of the Earth’s interior [1,2]. In order to reasonably interpret and extrapolate these observed phenomena of high EC anomalies, the in-situ measurements of laboratory-based EC of minerals and rocks are indispensable under controlled temperature and pressure conditions. Electrical conductivity is a temperature- and pressure-dependent intensive physical property of minerals, which largely depends on the physicochemical behavior of Earth’s and other terrestrial planets’ deep interior, and varies with depth [3,4]. In particular, EC is affected by several factors such as elements diffusion coefficients [5,6], electronic spin transition [7,8], crystallographic anisotropy [9,10], distribution of water and other volatile elements [11,12], partial melting [13,14], dehydration of minerals and rocks [15,16,17], pressure-induced structural phase transitions and metallization [18,19], etc.

Over the last twenty years, there has been a large number of research groups involved in measuring the EC of minerals in the upper-mantle and mantle transition zone using the electrical impedance spectroscopy (EIS) technique, combined with multi-anvil high-pressure devices. It is worth mentioning a few representative laboratories, such as the High-pressure Laboratory in the Department of Geology and Geophysics at Yale University, the Key Laboratory of High-temperature and High-pressure Study of the Earth’s Interior (HTHPSEI) in Institute of Geochemistry at Chinese Academy of Sciences, the Bayerisches Geoinstitut at University of Bayreuth, the Institute for Study of the Earth’s Interior at Okayama University, the Scripps Institution of Oceanography at University of California San Diego and the University Clermont Auvergne at the French National Centre for Scientific Research (CNRS).

In most of the models describing the upper mantle and the mantle transition zone, the main mineralogical composition consists of nominally anhydrous minerals (NAMs). The NAMs may accommodate a substantial amount of water, which widely exists as a form of hydrogen-related defects in the crystal structure of the minerals. The presence of trace hydrogen plays a crucial role in many pressure-dependent physicochemical properties and processes of minerals, such as electrical conductivity [3,4,11,12,20], diffusivity [21,22], plastic deformation [23,24], seismic wave attenuation [25,26], grain growth [27,28] and kinetic recrystallization [29,30]. In the early of 1990s, it was the first time that Karato put forward the hypothesis that the dissolved hydrogen can significantly enhance the EC of upper-mantle minerals and by using the Nernst-Einstein relation he calculated the EC of hydrous olivine [31]. As a hot topic in the research field of high-pressure mineral physics in the recent years, a series of experimental studies have been performed to clarify this hypothesis for the NAMS in the upper-mantle and transition zone (olivine: [4,12,32,33,34,35,36], pyroxene: [37], garnet [38,39,40], wadsleyite and ringwoodite [3,11,13]). About a decade later, Xu and his collaborators reported a series of EC measurements of mantle minerals, i.e., olivine, wadsleyite, ringwoodite and silicate perovskite with different content of alumina percentage, under high-pressure (HP) and high-temperature (HT) conditions [41,42,43,44,45,46]. By studying the in-situ EC of hydrous minerals at different water contents under high-*P* conditions, the functional relation between the EC and water content can be established, thereby providing constraints of the water content in the deep Earth’s interior.

Comprehensive considerations of laboratory-based EC measurements, geophysical field observations and theoretical-computational modelling, can be combined to shed light on the current structure of Earth. Although some possible causes of high conductivity anomalies in the upper mantle and mantle transition zone, such as the trace water of nominally anhydrous mineral, anisotropic hydrogen diffusion, partial melting and the presence of an interconnected impurity phase of high EC have been proposed in the recent years, some controversies still exist.

In the present work, the recent advances of the electrical transport properties of dominant minerals in the upper-mantle and mantle transition zone, i.e., olivine, pyroxene, garnet, ringwoodite and wadsleyite, under HP-HT conditions will be reviewed. The review paper is organized as follows: In Section 2, we briefly describe: (i) the well-established experimental technique of EIS used to perform conductivity measurements in various fields of material science and (ii) the main conduction mechanisms in mantle minerals. Section 3 reports on the HP-HT apparatuses used to simulate the mantle conditions for the investigation of minerals with different experimental approaches. A comprehensive data set of recently reported conductivity measurements is presented and discussed in Section 4. Finally, some important remarks on the evaluation of the mineral electrical conductivity are discussed in Section 5.

## 2. Electrical Conductivity of Minerals

### 2.1. Electrical Impedance Spectroscopy

The electrical properties of minerals and rocks have been studied extensively during the last decades and a wealth of published data is now available at HT and/or HP conditions [47,48,49,50]. Generally, the electrical conductivity, *σ* is frequency dependent and is therefore expressed as a complex function which is related to the other electrical-dielectric quantities, through the following relations:

(1) $$\sigma^{*}\left(\omega \right)=\sigma^{\prime }\left(\omega \right)-i\sigma^{\prime \prime }\left(\omega \right)=i\omega \varepsilon_{ο}\left(\varepsilon^{*}-1\right)$$

(2) $$\varepsilon^{*}\left(\omega \right)=\varepsilon^{\prime }\left(\omega \right)-i\varepsilon^{\prime \prime }\left(\omega \right)=C\left(\omega \right)\frac{d}{\varepsilon_{ο}\pi r^{2}}-i\frac{1}{\omega R\left(\omega \right)}\cdot \frac{d}{\varepsilon_{ο}\pi r^{2}}$$

and:

(3) $$Z^{*}\left(\omega \right)=Z^{\prime }+iZ^{\prime \prime }=\frac{R}{1+{\left(\omega RC\right)}^{2}}-i\frac{\omega R^{2}C}{1+{\left(\omega RC\right)}^{2}}$$

where primed quantities denote the real part and the double primed the imaginary part of the complex EC $\sigma^{*}\left(\omega \right)$, dielectric permittivity $\varepsilon^{*}\left(\omega \right)$ and complex impedance $Z^{*}\left(\omega \right)$ [49]. The angular frequency is denoted by *ω*, $\varepsilon_{ο}$ is the permittivity of the vacuum and $i=\sqrt{-1}$. Equations (2) and (3) stand for a parallel combination of an ohmic resistance *R* and a capacitor *C*, which is the case of measurements carried out with an impedance analyzer operating on the two-electrodes configuration, with a separation distance *d* and radius *r* of the cylindrical electrodes.

The advantage of measuring the electrical impedance of a sample in a broad range of frequencies, usually between a few mHz to several MHz depending on the type of the analyzer, lies in the fact that from the recorded spectra we can easily distinguish the different contributions to the overall EC, namely, grain interior, grain boundaries and electrodes effects. In this way, only the intrinsic conductivity of the bulk of the mineral will be considered and thus, we will avoid any possible errors resulting from the unwanted contributions. This can be achieved by plotting the recorded data in Cole-Cole plots of impedance, i.e., the imaginary part of impedance versus its real part. In the simplest case of a series combination of *R-C* elements connected in parallel (according to Equation (3)), the different contributions are indicated by separated and/or overlapping semicircles. However, in the case of natural samples such as minerals or rocks in different forms (single crystal, polycrystalline or powder), the ideal semicircle is reduced to a depressed one and the capacitance in Equation (3) is replaced by a constant phase element (CPE), according to the following definition:

(4) $$Z_{CPE}=\frac{1}{Q{\left(i\omega \right)}^{n}}=\frac{1}{Q\omega^{n}}\left[cos\left(-n\frac{\pi }{2}\right)+isin\left(-n\frac{\pi }{2}\right)\right]$$

where the parameters *Q* and *n* are calculated from the fitting of the experimental data [51]. The resistance *R* of the sample results from the intersection of the fitted arc with the real axis ($Z^{\prime }$) and therefore, the conductivity is calculated from the relation, $\sigma =d/\left(\pi r^{2}R\right)$.

**Figure 1:**
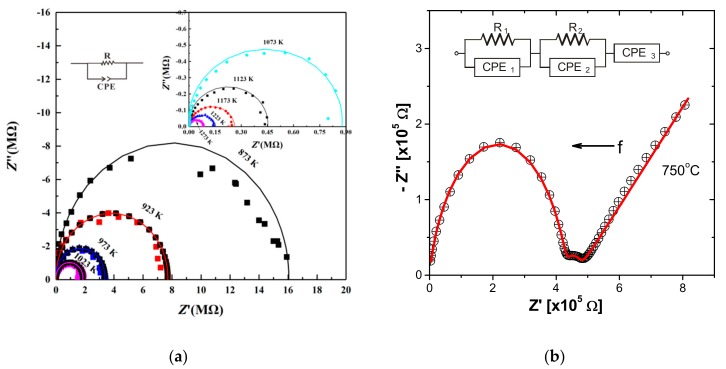
Cole-Cole plots of impedance (−*Z”* vs. *Ζ’*) at two different cases. Solid colored lines represent the fitting of experimental data to an equivalent circuit. (**a**) Dry olivine aggregates measured at elevated temperatures (873–1273 K) and pressure of 2 GPa [52]. (**b**) Diatomite sample measured at 750 °C, in the frequency range 10^−2^–10^6^ Hz. The contributions of grains interior, grain boundaries and electrode effects are clearly shown as two depressed semicircles accompanied with a tail in the low frequency range. The equivalent circuit is also shown (data from [51]).

Examples of Cole-Cole plots of complex impedance spectra of mineral and rock samples, are depicted in Figure 1a,b. Figure 1a shows the electrical impedance measurements of magnetite-free dry olivine aggregates [52] carried out in the frequency range from 10^−1^ to 10^6^ Hz, at 2.0 GPa and at elevated temperatures (873–1273 K). In this case, an equivalent circuit comprising a resistance *R* in parallel connection to a CPE is sufficient to describe the electrical response of the mineral, under the specific measured conditions. A deviation of the experimental data measured at 873 K from the arc-shaped fitting model, suggests that at this temperature an additional contribution might exist, which however disappears at higher temperatures. In the case of the impedance measurements of a diatomite sample shown in Figure 1b, the overall electrical response is modeled by a series combination of two parallel *R*-CPE circuits and a single CPE, indicating the existence of three distinct contributions, i.e., grains interior, grain boundaries and electrodes polarization effects [51].

In most cases, dc-conductivity is calculated from the recorded impedance spectra and its temperature and pressure dependence for a single conduction mechanism is described by the well-known Arrhenius law:

(5) $$\sigma \left(T,P\right)=\sigma_{o}exp\left(-\frac{\Delta E+P\Delta V}{RT}\right)$$

where $\sigma_{o}$ is the pre-exponential factor, Δ*E* is the activation energy, Δ*V* is the activation volume and *R* is the gas constant [4].

It is worth mentioning that the activation volume is an important point defect parameter as it is related to the lattice distortion caused by the formation or/and the transportation of charge carriers in conduction and diffusion processes. Actually, in the case of a single conduction mechanism in a mineral, the EC is related to the diffusion of the electric charge carrier through the Nernst-Einstein equation. The sign of Δ*V* depends on the lattice expansion or shrinkage caused when the charge carrier is transferred between two consecutive lattice positions. In this sense, models that describe the self- and hetero-diffusion in minerals are very important for a theoretical approach giving insights to the transport properties of conduction and diffusion [53]. The aforementioned issues will be discussed further in the last section.

### 2.2. Electrical Conduction Mechanisms in Mantle Minerals

When different conduction mechanisms contribute independently to the overall electrical response of a mineral, the total EC is expressed as a sum of the contributions of the different charge carriers:

(6) $$\sigma =\underset{i}{\sum }q_{i}n_{i}\mu_{i}$$

where $\mu_{i}$ express the mobility of the *i*-th charge carrier with an effective charge $q_{i}$, and $n_{i}$ denotes its concentration [4]. The transport of each charge carrier is a thermally activated process, which dominates at a certain temperature range and is characterized by an activation energy, Δ*E*. Consequently, different charged species having different activation energies can be distinguished from each other as they correspond to different slopes in an Arrhenius plot (refer to Equation (5)) that can appear if EC is measured over a wide temperature range.

In the case of mantle minerals such as silicates (olivine and its high-*P* polymorphs) and oxide minerals (ferropericlase, (Mg,Fe)O), electrical conduction is mainly attributed to ionic diffusion of Fe^2+^, Mg^2+^ or protons, and to hopping of electrons or electron holes [50,54].

Ionic conduction takes place via diffusion of Fe or Mg ions through vacancy sites in the mineral lattice which requires large activation energy and therefore dominates at high temperatures. Additionally, in H-bearing minerals, the diffusion of hydrogen (proton) or H-related defects may have a significant contribution to the electrical conduction. In this case, due to the high mobility of protons, the activation energy is smaller [54].

In Fe-bearing minerals, the existence of ferric ions (Fe^3+^) in ordinary cation sites ($Fe_{M}^{•}$ in Kröger-Vink notation, where M denotes Fe or Mg sites) results to the creation of electron holes that contribute to the electrical conduction through the exchange of electron charge between ferrous and ferric irons. However, in insulating materials such as minerals, the hopping of the electron charge with a reduced mobility causes the distortion of the surrounding lattice. The latter mechanism is known as the hopping of small polaron, where “small” refers to the local lattice distortion, as opposed to the extended spatial deformation over many lattice sites where the polaron is considered as large [49,50].

## 3. High-Pressure Apparatuses for Conductivity Measurements of Minerals and Rocks of Upper Mantle and Mantle Transition Zone

**Figure 2:**
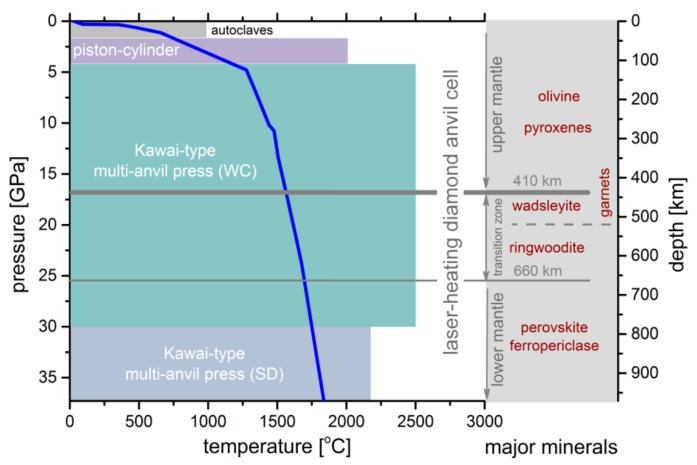
The pressure and temperature ranges of different types of apparatuses (denoted with different colors) in a *T-P* profile of the Earth (blue line), according to the Preliminary Reference Earth Model (PREM) [55]. The major minerals of the mantle up to the depth of 900 km have been noted.

In order to perform the EC measurements of minerals and rocks under HP-HT conditions, many different high-pressure apparatuses have been designed and developed in the past, such as autoclaves, piston-cylinders, multi-anvil presses and diamond anvil cells. A schematic diagram of the *P*-*T* operating limits of the above devices is shown in Figure 2. 

Among all these high-pressure equipments, the multi-anvil press has unique advantages for performing electrical conductivity experiments. Firstly, the relatively large space of sample chamber can accommodate a complex sample assemblage for EC measurements. Secondly, it can provide an efficient hydrostatic pressure environment and a stable high temperature range. In light of the temperature and pressure ranges of the upper-mantle and mantle transition zone regions, there are two representative multi-anvil presses, namely, YJ-3000t and Kawai-1000t that have been adopted to measure the EC of minerals and rocks under HP-HT conditions till now. These two types of devices operating in similar *P*-*T* ranges are presented below. An electrical cell assembly for performing EC measurements in multi-anvil apparatuses was recently made available by the Consortium for Material Properties Research in Earth Sciences (COMPRES) [56] and is also described below.

### 3.1. YJ-3000t Multi-Anvil Press

**Figure 3:**
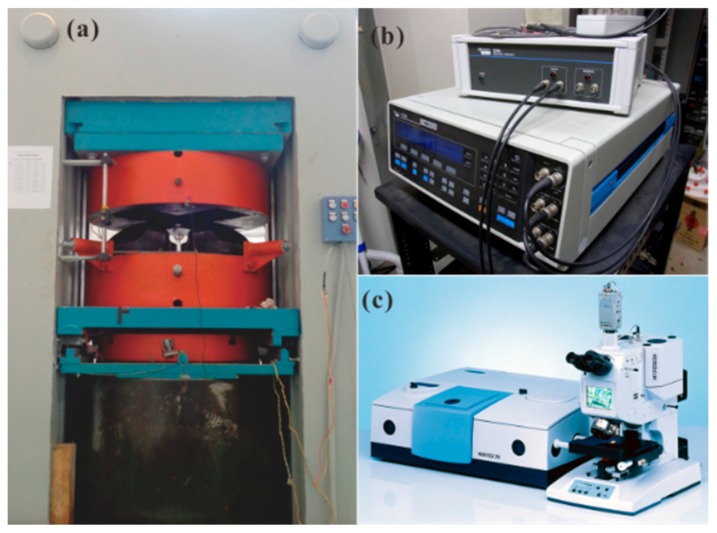
Experimental platform of HTHPSEI for electrical conductivity measurements of minerals and rocks at HP-HT conditions. (**a**) The pressure-generated apparatus of the YJ-3000t multi-anvil press; (**b**) the Solartron-1260 Impedance/Gain-phase analyzer used for measuring the complex impedance of the sample; (**c**) the Vertex-70v vacuum FTIR analyzer used to determine the water content before and after high-pressure EC measurements.

Almost half a century ago, the YJ-3000t multi-anvil press was successfully installed and operated by Xie and his coworkers in the Key Laboratory of HTHPSEI, Institute of Geochemistry, Chinese Academy of Sciences, Guiyang, Guizhou, China. Following the first study on the EC of fayalite carried out by Xie et al. [57] under HP-HT conditions, a large amount of in-situ EC results on minerals, rocks, pure water, and saline solution systems have been published the last 35 years, using this high-pressure apparatus [58,59,60,61,62,63,64,65,66,67,68,69,70,71,72,73,74,75]. Figure 3 shows the experimental measurement platform of HTHPSEI used for performing the EC measurements of minerals and rocks at HP-HT conditions. It is composed of three main counterpart pieces of equipment, namely, (a) the pressure-generated apparatus of the YJ-3000t multi-anvil press; (b) the Solartron-1260 Impedance/Gain-phase analyzer operating in the two-electrodes configuration for complex EIS measurements in the frequency range 10^−2^ Hz–10^6^ Hz; and (c) the Vertex-70v vacuum Fourier-transform infrared spectroscopy (FT-IR) analyzer used for determining the water content before and after the high-pressure EC measurements. All potential parameters affecting the EC of minerals and rocks in the deep Earth crust and upper mantle (i.e., temperature, pressure, frequency, oxygen fugacity, water content, iron content, crystallographic anisotropy, grain boundary state, content of alkali metallic elements, et al.) have been thoroughly explored using this in-situ HP measurement platform.

**Figure 4:**
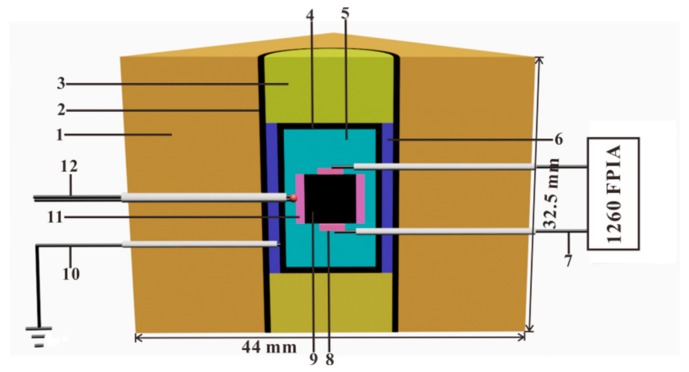
Experimental assemblage for EC measurements in the YJ-3000t multi-anvil press. (1) pressure medium of cubic pyrophyllite (baked at 973 K); (2) three layers stainless steel heater; (3) symmetric pyrophyllite block (baked at 1173 K); (4) metallic shielding case made of Ni, Fe, Re or Mo foil; (5) MgO insulation tube; (6) Al_2_O_3_ insulation tube; (7) lead wire of metallic electrode and Al_2_O_3_ insulation tube; (8) two symmetric buffer electrodes; (9) sample; (10) electric grounding; (11) solid oxygen buffer; (12) thermocouple and Al_2_O_3_ insulation tube.

By virtue of the YJ-3000t multi-anvil press, the representative measurement assemblage for measuring the EC of upper-mantle minerals and rocks under controlled oxygen fugacities, using the EIS technique, was designed by Dai and his coworkers [76,77,78], as illustrated in detail in Figure 4. During the EC measurements, the oxygen fugacity of sample chamber can be efficiently monitored and adjusted by changing the selective metallic type in two symmetric disc-shaped metallic electrodes (e.g., Ni, Mo, Re, Fe, etc.), its corresponding metallic tubes with the same kind of the metal-containing and metallic oxide (e.g., Ni–NiO, Mo–MoO_2_, Re–ReO_2_, Fe–FeO, Fe–Fe_3_O_4_, etc.) and the Faraday shielding case. High pressure is generated by 6 cubic anvils made of tungsten carbide (WC) with each edge length of 23.4 mm, which can provide a maximum pressure of less than 10.0 GPa. The cubic pyrophyllite (32.5 × 32.5 × 32.5 mm^3^) was chosen as the pressure-transmitting medium, and some representative Al_2_O_3_ and MgO ceramic sleeves were adopted to surround the sample so as to supply a sufficient insulation environment. The heater was made up of three-layer stainless steel slices with each individual layer thickness of ~1.7 mm. The experimental temperature was monitored by NiCr–NiAl, W5%Re95%–W26%Re74% and Pt–Pt90%Rh10% thermocouples, with an error of ±5 K.

In addition to the in-situ EC measurements, it has recently become possible to measure some other high pressure-dependent physical properties of minerals and rocks by using the YJ-3000t multi-anvil press, such as the ultrasonic elastic wave velocity, thermal conductivity, thermal diffusivity, kinetics of grain growth, etc. [79,80,81,82,83,84,85,86,87]. These additional capabilities make the system a state-of-the-art experimental tool for performing HP-HT measurements in many areas of materials science.

### 3.2. Kawai-1000t Multi-Anvil Press

**Figure 5:**
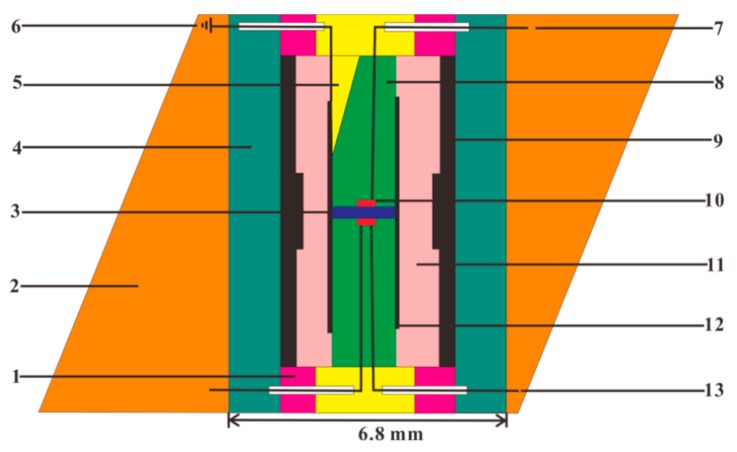
Experimental assemblage for EC measurements in the Kawai-1000t multi-anvil press: (1) metallic Mo ring; (2) MgO octahedral pressure medium with its edge length of 14 mm; (3) sample; (4) zirconia; (5) Al_2_O_3_ cement; (6) electric grounding; (7) lead wire of metallic electrode and Al_2_O_3_ insulation tube; (8) insulation tube made of four hole alumina; (9) heater of lanthanum chromite; (10) two symmetric buffer electrodes; (11) MgO insulation tube; (12) metallic shielding case made of Ni, Fe, Re or Mo foil; and (13) thermocouple and Al_2_O_3_ insulation tube.

A typical sample assembly for high-pressure EC measurements using the Kawai-1000t multi-anvil high-pressure apparatus installed in Department of Geology and Geophysics at Yale University (New Haven, CT, USA) [3,11,12,32,33,34,35,37,38] is displayed in Figure 5. In this type of multi-anvil apparatus, pressure is generated by eight cubic WC anvils (26 × 26 × 26 mm^3^) with the 3 to 18 mm truncated edge length, depending on the pressure. High-pressure phase transitions of some representative engineering materials (ZnS, ZnTe, GaP, etc.) and minerals (coesite, olivine, wadsleyite, Bridgmanite, et al.,) have been chosen to calibrate the pressure of sample chamber in the Kawai-1000t multi-anvil high-pressure apparatus. Some fundamental parts including the MgO octahedral pressure medium with the percentage of 5% doped Cr_2_O_3_, Al_2_O_3_ insulation sleeve, two symmetric disk-shaped metallic electrodes and metallic foil shielding are employed to perform the EC measurements at HP conditions. Different types of heaters are available, according to the target temperature and the requirement of sample assemblage, such as graphite, lanthanum chromite, titanium diboride, rhenium slice, etc. The W5%Re95%–W26%Re74% and Pt–Pt90%Rh10% thermocouples are adopted to measure the temperature during the EC measurements. The uncertainties of the temperature and pressure are 10 K and 0.5 GPa, respectively.

### 3.3. The Electrical Cell by COMPRES

Recently, an electrical sample cell was developed by Pommier and Leinenweber [56] and became available through COMPRES, in order to ensure high-quality and reproducible EC measurements under HP-HT conditions. This electrical cell can be combined with a multi-anvil assembly and has been tested by using rhenium and graphite furnaces and different types of electrodes (Mo and Fe), and calibrated at pressures up to 10 GPa and temperatures up to 2273 K. An advantage of this electrical cell lies in the choice of using 2- or 4-electrodes configuration. The latter setup is achieved by using the wires of the two W-Re thermocouples, preventing the unwanted contribution of charge accumulation to the electrodes, which occurs at low frequencies of the applied ac-voltage. Furthermore, in the 4-electrodes configuration, the use of the two thermocouples allow the detection of a thermal gradient across the sample, thereby avoiding uneven heating, which results in the chemical heterogeneity of the tested sample [88,89].

## 4. Electrical Conductivity of Major Minerals in the Upper Mantle and Mantle Transition Zone

The main factors influencing the electrical transport properties of minerals and rocks, i.e., temperature, pressure, oxygen fugacity, water content, iron content, grain boundary state, graphite content, magnetite content, anisotropy and partial melting have been explored in detail, in the recent years. Due to the neglected effect of the crucial water content on the EC of the dominant mantle minerals studied by other research groups, in the following we mainly focus on the high-pressure conductivity results from the Institute of Geochemistry at Chinese Academy of Sciences and the Department of Geology and Geophysics at Yale University.

### 4.1. Electrical Conductivity of Olivine

Olivine is a dominant rock-forming silicate and nominally hydrous mineral in the Earth crust and upper mantle, which can be stabilized over a wide temperature and pressure range, in the depth range of 5 to 410 km. The EC of olivine single crystal is anisotropic and highly sensitive to pressure, oxygen fugacity, water content and iron content. In an early study, Roberts and Tyburczy [48] reported the electrical properties of polycrystalline olivine compacts over a broad frequency range. They investigated the role of porosity and induced microfracturing due to the thermal expansion anisotropy of olivine, in the measured conductivity. They concluded that, for their measured samples of 2–8% vol. inter- and intra-granular porosity, the above features have little or no effect to the overall electrical properties. On the basis on these findings, subsequent investigations on the electrical properties of olivine and other mantle minerals do not include the effects of porosity and micro-cracking. The pressure influence on the EC of San Carlos olivine Mg_1.8_Fe_0.2_SiO_4_ single crystal was investigated by Xu et al. [41] using the EIS technique in the multi-anvil high-pressure apparatus. The conductivity measurements were carried out in the temperature range of 1273–1673 K, pressure range of 4–10 GPa and the Mo–MoO_2_ oxygen buffer, within the frequency range of 10^−1^–10^6^ Hz. The experimental data were fit to the Arrhenius relation with the pre-exponential factor of ~200 S/m, Δ*E* of 144.7 kJ/mol and Δ*V* of 0.6 cm^3^/mol [46]. A negative pressure dependence on the EC of the olivine single crystal was observed and the hopping conduction mechanism of small polaron between the ferrous and ferric iron was proposed. However, in all of their published results on the EC of mantle minerals, Xu et al., did not provide any information on the water content of the measured samples [41,42,43,44,45,46].

The first theoretical prediction for the effect of water on the EC of mantle olivine was put forward by Karato [31] on the basis of the Nernst-Einstein equation, who stated that the trace hydrogen of nominally hydrous mineral plays a crucial role in the EC of olivine in the upper mantle. In order to test this hypothesis, the EC of hydrous synthetic olivine was measured for the first time by Wang et al. [12] at conditions of 4.0 GPa, 873–1273 K and water content of 100–800 ppm, using the Kawai-1000t multi-anvil press (Key Laboratory of High-Temperature and High-Pressure Study of the Earth’s Interior, Institute of Geochemistry, Chinese Academy of Sciences, Guiyang, China) and the Solarton-1260 EIS analyzer (Schlumberger, Houston, TX, USA). They suggested the model of the ionization reaction in hydrous olivine for the free proton-dominated conduction mechanism, as follows:

(7) $${\left(2H\right)}_{M}^{\times }=H_{M}^{\prime }+H^{•}$$

where, in the Kröger-Vink notation, ${\left(2H\right)}_{M}^{\times }$ stands for the two hydrogens of crystalline lattice in the *M* site, $H_{M}^{\prime }$ stands for the hydrogen vacancy of *M* site, $H^{•}$ stands for the free proton and *M* stands for either Mg or Fe.

**Figure 6:**
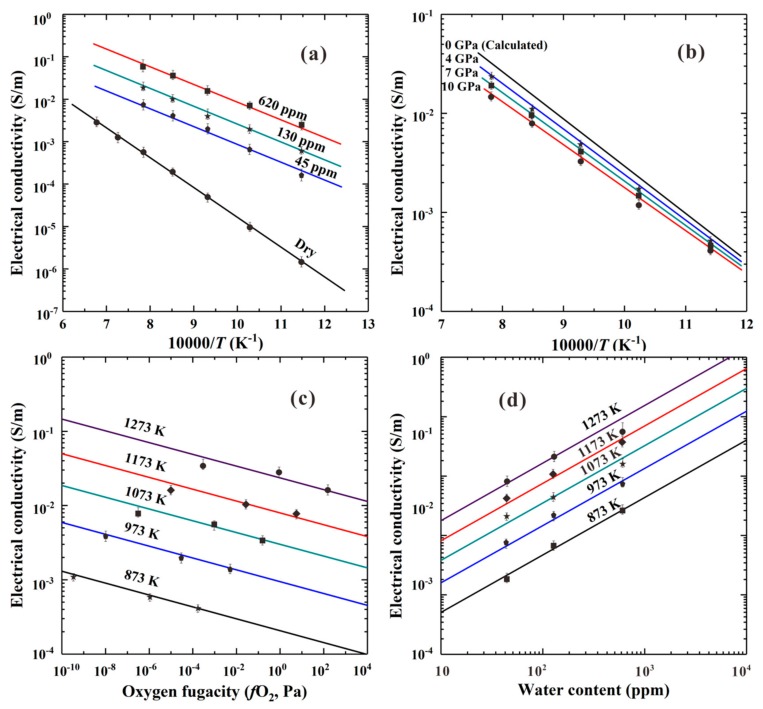
The influence of (**a**) temperature and water content, (**b**) temperature and pressure, (**c**) oxygen fugacity and temperature, and (**d**) water content and temperature on the EC of hydrous olivine aggregates under HP-HT conditions (873–1473 K and 4–10 GPa). Oxygen fugacity is controlled by three different solid-state oxygen buffers, increasing along Ni–NiO, Mo–MoO_2_ and Re–ReO_2_ (Reproduced with permission from Dai and Karato, Phys. Earth Planet. Inter.; published by Elsevier, 2009 [32,33,34]).

Furthermore, the electrical conductivities of the olivine single crystal, polycrystalline and sintered synthetic polycrystalline hydrous olivine aggregates have been extensively investigated in recent years [32,33,34,35]. The effects of temperature, pressure, oxygen fugacity, iron and water content on the EC of olivine single crystal and polycrystalline compacts were studied under HP-HT conditions is shown in Figure 6.

For olivine aggregates with a constant iron content, *X*_iron_ = 0.412 (molar ratio, Fe/(Fe+Mg)), we observe that by increasing the water content from 45 to 620 ppm, EC increases by an order of magnitude, retaining the same activation enthalpy, Δ*H* = 80 kJ/mol (refer to Figure 6a,d). This activation enthalpy is lower than that of the dry olivine sample with the same iron content, Δ*H* = 136 kJ/mol [32]. The effect of pressure on the EC of hydrous olivine aggregates in the temperature range 873–1273 K is depicted in Figure 6b. EC decreases with increasing pressure up to 10 GPa, affecting both activation enthalpy and pre-exponential factor, which also decrease (refer to Table 1) [34]. In all the previous cases, a single slope in the Arrhenius plots is observed in the measured temperature range (873–1473 K) suggesting the operation of a single conduction mechanism. The effect of oxygen fugacity on the H-assisted EC of hydrous (280 ppm) polycrystalline olivine measured at 873–1273 K and 4.0 GPa is shown in Figure 6c, where a negative dependence is observed at all the measured temperatures. The latter observation has been attributed to a hybrid model of EC where a transition from the free-proton conduction to a mechanism associated with two protons at M-site can take place [33].

Dai and Karato [32] reported that the content of iron and hydrogen in olivine can enhance the EC of anhydrous and hydrous samples under HP-HT conditions, respectively. The EC of hydrous Fe-bearing silicate minerals with different contents of iron and hydrogen has been expressed as:

(8) $$\sigma =\sigma_{iron}+\sigma_{hydrogen}$$

where $\sigma_{iron}$ stands for the EC due to the small polaron hopping conduction of anhydrous specimen, and $\sigma_{hydrogen}$ denotes the EC due to the hydrogen-related defect of hydrous sample. The total conductivity of both conduction mechanisms, has been fitted using the following equation:

(9) $$\sigma =\sigma_{01\left(\mathrm{iron}\right)}X_{\mathrm{iron}}^{\;\;\;\;\;\;i}f_{O_{2}}^{q_{1}}\exp\left(-\frac{\Delta H_{iron}}{kT}\right)+\sigma_{01\left(\mathrm{hydrogen}\right)}X_{\mathrm{hydrogen}}^{\;\;\;\;\;\;\;\;\;\;\;\;\;\;\;\;\;\;i}f_{O_{2}}^{q_{2}}C_{w}^{r}\exp\left(-\frac{\Delta H_{\mathrm{hydrogen}}}{kT}\right)$$

where $\sigma_{01\left(\mathrm{iron}\right)}$ and $\sigma_{01\left(\mathrm{hydrogen}\right)}$ stand for the pre-exponential factors of iron- and water-bearing sample, respectively; the parameters *X*, $f_{O_{2}}$, *C_W_* and Δ*H* stand for iron (or hydrogen) content, oxygen fugacity, water content and activation enthalpy of sample, respectively; all of *i*, *q*_1_, *q*_2_, *r* and *k* stand for constants [32,33].

Dai and Karato found that as the weight fraction of ferrous iron was increasing from 0.214 to 0.637, the EC of anhydrous olivine also increased, while, Δ*H* of sample reduced accordingly, from 148 to 121 kJ/mol [32]. In contrast, the EC of hydrous olivine had a weak dependence on iron content and Δ*H* was almost independent (80–88 kJ/mol) over the temperature range of 873–1473 K at 4.0 GPa. Therefore, the influence of iron content on the EC of hydrous olivine is very feeble over the whole temperature range, for the range of water content, 45–620 ppm.

The observed dependence between the EC of olivine and oxygen fugacity, expressed through the *q*-exponent (refer to Equation (9)), was used to efficiently distinguish the species of charge carrier and its electric transport, i.e., small polaron ($Fe_{\mathrm{Mg}}^{•}$) and hydrogen-related defects (free proton (*H^•^*), one hydrogen vacancy at M-site $(H_{M}^{\prime }$) and two protons at *M*-site (${\left(2H\right)}_{M}^{x}$*)*, respectively [33].

The pressure dependence on the EC of dry and water-rich mantle olivine, was systematically studied by Xu et al. [46], and Dai and Karato [34], within the corresponding upper-mantle pressure range, 4.0–10.0 GPa. A negative pressure dependence of EC of dry and wet olivine was observed, and a positive activation volume (Δ*V* = 0.6 cm^3^/mol) for dry sample was obtained, whereas, a negative value of activation volume (Δ*V* = −0.86 cm^3^/mol) was confirmed for the hydrous olivine. This observation is possibly related to the different pressure dependences of EC of olivine single crystals with various water contents and the electrical conduction mechanism at high-*P*.

**Figure 7:**
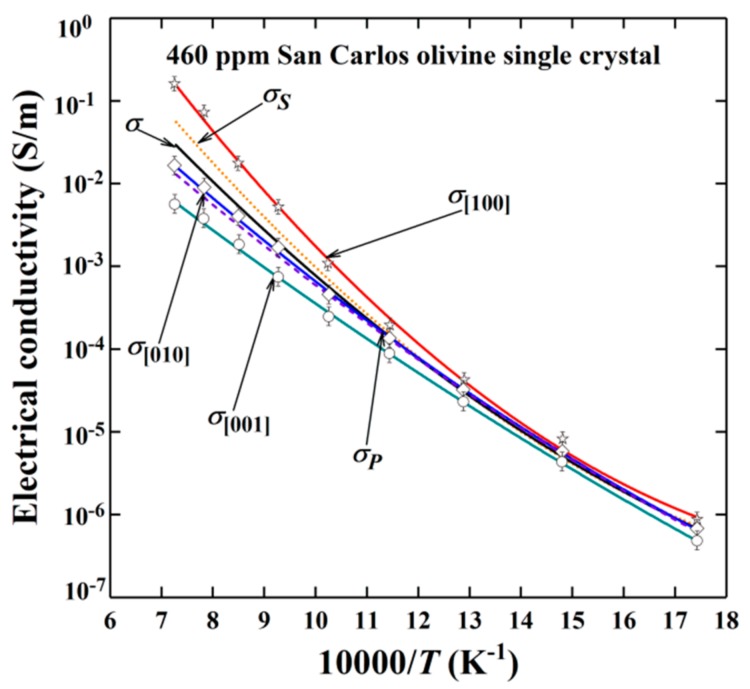
The anisotropic EC of San Carlos olivine single crystals measured at 4.0 GPa over a broad temperature range (573–1373 K). Three different average schemes of series (*σ*_s_), parallel (*σ*_p_) and effective medium models (〈σ〉) were applied. See text for details. (Reproduced with permission from Dai and Karato, Earth Planet. Sci. Lett; published by Elsevier, 2014 [35]).

The EC of hydrated olivine single crystals along the three dominant crystallographic directions ([100], [010] and [001]) has been reported by Dai and Karato [35] under conditions of a broad temperature range of 573–1373 K at 4.0 GPa (Figure 7). In the low temperature range (573–900 K), the EC of olivine single crystal with a low Δ*H* (74 kJ/mol) shows a feeble anisotropy, which is in good agreement with previously obtained conductivity results [12,93,94,95]. However, within the high temperature range (T > 1000 K), the EC of sample exhibiting a higher activation enthalpy (~130–150 kJ/mol) is obviously anisotropic, which is consistent with the results of high-temperature H–D inter-diffusion in olivine [96].

In order to precisely estimate the EC of randomly oriented polycrystalline aggregates from the anisotropic EC results of olivine single crystals along [100], [010] and [001] crystallographic orientations, three different average schemes of series (*σ*_s_), parallel (*σ*_p_) and effective medium models (⟨σ⟩) were proposed (refer to Figure 7), as follows [35]:

(10) $$\sigma_{S}=\left(\sigma_{\left[100\right]}+\sigma_{\left[010\right]}+\sigma_{\left[001\right]}\right)/3$$

(11) $$\sigma_{P}=3{\left(\frac{1}{\sigma_{\left[100\right]}}+\frac{1}{\sigma_{\left[010\right]}}+\frac{1}{\sigma_{\left[001\right]}}\right)}^{-1}$$

(12) $$\langle \sigma \rangle =\left[\sigma_{S}+\sigma_{P}+\sqrt{{\left(\sigma_{S}+\sigma_{P}\right)}^{2}+32\sigma_{S}\sigma_{P}}\right]/8$$

In comprehensive considerations of geophysical field observations and geochemical models, the acquired EC results revealed that the high and highly anisotropic EC at the corresponding asthenospheric temperature and pressure conditions is reasonably explained by the high water content in the region of asthenosphere (100 ppm). On the other hand, the influence of the interconnected high conductive impurity phases (graphite, magnetite, sulfide impurity, etc.) on the EC of olivine has been also explored in detail [52,89,96,97,98]. An alternative approach to the highly anisotropic EC of the lithosphere-asthenosphere boundary was recently proposed by Pommier et al., who studied the effect of melt in initially deformed minerals and rock samples [88]. Specifically, Pommier et al., performed EC measurements in deformed olivine aggregates and sheared partially molten rocks, at asthenospheric pressure (~3 GPa) and temperatures up to 1479 K. They found that the EC in the shear direction of the deformed olivine samples is one order of magnitude higher as compared to the undeformed samples while, it increases by two orders of magnitude in melt-bearing layered samples of olivine.

### 4.2. Electrical Conductivity of Pyroxene

Pyroxene is the secondary primary abundant component of the upper mantle and its mineralogical composition corresponds approximately to 20–40% by volume of the upper mantle. Therefore, pyroxene and its high-pressure polymorphs can provide an important constraint on the bulk EC of the upper mantle. Previous results on the aluminum-bearing saturated water solubility for anisotropic enstatite indicated that the orthopyroxene can dissolve an abnormally high amount of structural water than olivine in the Earth’s asthenosphere region [90,99], hinting that pyroxene plays a crucial role in explaining the high conductivity anomaly in the shallow upper mantle conditions. Thus, a large amount of EC results has been reported for orthopyroxene and clinopyroxene, using the EIS measurement technique under HP-HT conditions.

Electrical conductivity measurements of 2.89 wt% alumina-bearing San Carlos orthopyroxene [(Mg_0.92_Fe_0.08_)SiO_3_] were carried out by Xu and Shankland [44], using the multi-anvil press and EIS technique under the pressure range of 5–21 GPa, temperatures of 1273–1773 K and controlled oxygen fugacity of the Mo–MoO_2_ solid buffer. By virtue of the fitted Arrhenius relations from the in-situ EC results, the phase transitions from orthopyroxene to clinopyroxene to ilmenite and garnet system imposed constraints on the EC of the mantle.

Dai et al. [100] measured the EC of dry orthopyroxene at 1.0–4.0 GPa and 1073–1423 K under controlled oxygen partial pressure conditions. Four solid oxygen buffers (e.g., Ni–NiO, Fe–Fe_3_O_4_, Fe–FeO and Mo–MoO_2_) were selected to control the oxygen fugacity during the EC measurements. At the pressure of 2.0 GPa, the functional modelling between the EC of the dry sample and the variation of oxygen fugacity was successfully established.

**Figure 8:**
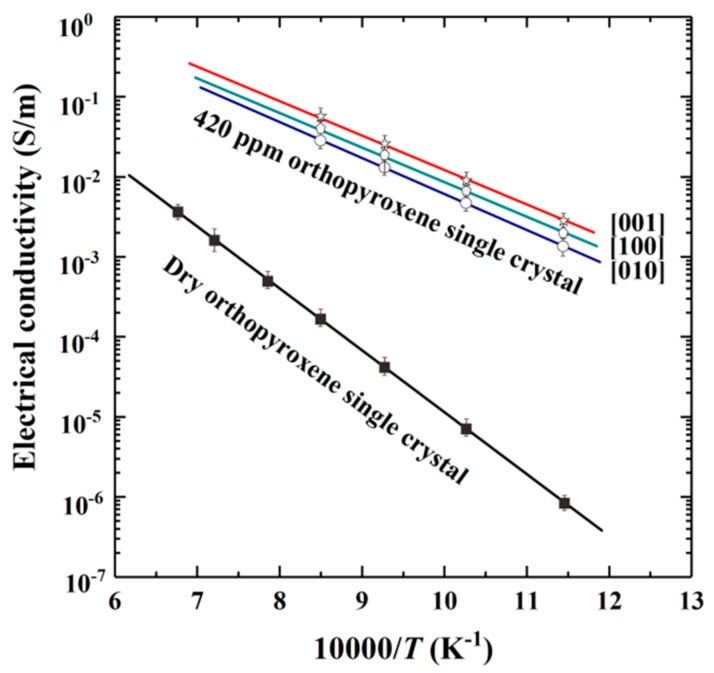
The electrical conductivity of hydrous orthopyroxene single crystals along [100], [010] and [001] crystallographic directions at conditions of 873–1273 K and 8 GPa. The EC of anhydrous sample is also included (modified from Dai and Karato [37]).

Dai and Karato performed EC measurements on two oriented orthopyroxene single crystals from the Stuttgart region in Germany and the Han Nuoba region in eastern China [37]. The water content of orthopyroxene was accurately determined by FT-IR spectroscopy, before and after the EC measurements. The Arrhenius plot of EC of anhydrous and hydrous orthopyroxene single crystals (water content, 420 ppm) along the three main crystallographic orientations ([001], [100] and [010]) is depicted in Figure 8, where it is evident that: (i) the EC of the hydrous orthopyroxene single crystal is higher than that of the anhydrous sample and (ii) the influence of anisotropy on the activation enthalpy and the pre-exponential factor of the hydrous orthopyroxene single crystal is rather weak (refer also to Table 1). A good correlation between the EC and temperature according to an Arrhenius relation implies that only one individual conduction mechanism can operate under the conditions of 8 GPa, 873–1473 K and Mo–MoO_2_ oxygen buffer. Dai and Karato suggested that the main conduction mechanisms for anhydrous and hydrous Fe-bearing orthopyroxene samples are the hopping of small polaron and the free proton diffusion, respectively.

In addition, Yang et al. [101] and Schlechter et al. [102] measured the EC of hydrous orthopyroxene containing various amounts of hydrogen, aluminum and iron. The functional relationship of EC with the water content (0, 40, 100 and 285 ppm) was established by Yang et al. [101] under conditions of 0.6–1.2 GPa and 573–1273 K in an end-loaded piston cylinder apparatus. They reported that the EC of hydrous orthopyroxene can explain the high EC anomaly in the lower crust regions, especially where its main rock outcrop is constituted mostly by granulite.

The electrical conductivity of dry diopside single crystals along different crystallographic directions was reported by Dai et al. [103] under conditions of 1.0–4.0 GPa, 1073–1373 K and Ni–NiO oxygen buffer, using the YJ-3000t multi-anvil press and the Solartron-1260 EIS analyzer. A phenomenon of feeble anisotropic EC in dry clinopyroxene single crystals was also observed along the [001], [100] and [010] crystallographic orientations, which is similar to the results for the EC of hydrous orthopyroxene single crystals [102]. The complex EC of polycrystalline augite with different grain sizes (Spec: ~5–63, 63–160 and 160–250 μm) were measured by Yang and Heidelbach [104] under conditions of 1.0 GPa and 773–1273 K in the piston-cylinder high-pressure apparatus. Compared with the EC data of clinopyroxene single crystal, a negligible influence of grain size on the EC was observed. Subsequently, a series of results for the effect of water content on the EC of hydrous clinopyroxene were reported by Yang [90], Yang et al. [105], Zhao and Yoshino [91], and Liu et al. [106], in order to explain the high conductivity anomaly of magnetotelluric field observations in the representative regions of the lower continental crust, the uppermost mantle, the Eastern Pacific rise region, the ultrahigh-pressure metamorphic belt of Dabieshan and the Tibet Plateau.

### 4.3. Electrical Conductivity of Garnet

As a major rock-forming mineral, garnet is able to stabilize over a broad temperature and pressure range of the Earth’s interior, from the mid-lower crust to the top of lower mantle. In the light of its structural stability and its complex chemical composition, garnet is widely distributed over the entire deep mantle region, as compared to some typical mantle rock-forming minerals, i.e., olivine, clinopyroxene, orthopyroxene, wadsleyite, etc. Therefore, the HP-HT research on the EC of garnet is essential to establish the laboratory-based electrical conductivity-depth profile and explain the high EC anomaly in the deep Earth’s interior.

ECs of majorite garnet with two different chemical compositions of pyrolite (pyrolite minus olivine) and mid-ocean ridge basalt have been reported by Yoshino et al. [107] under the mantle transition zone conditions of 18–23 GPa and 900–2000 K. Within the lower temperature range of 1000–1600 K, the relatively high activation energy of 135 kJ/mol indicates that the main charge carrier of majorite garnet is transported by the hopping of small polaron. However, at higher temperatures (1600–2000 K), the dominant charge carrier is migrated by the ionic diffusion.

**Figure 9:**
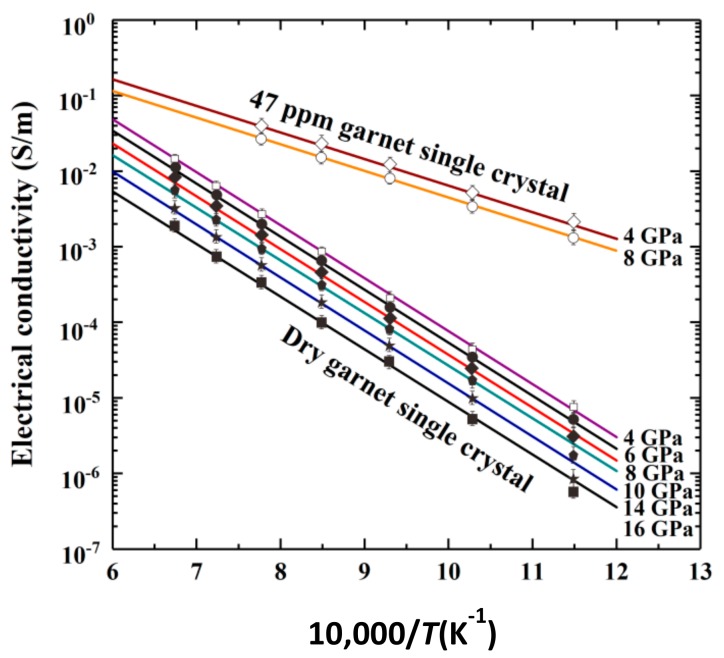
The effect of the pressure on the EC of anhydrous and hydrous pyrope-rich garnet single crystals (~Py_73_-Alm_14_-Grs_13_) in the temperature range of 873–1473 K. The abbreviations Alm and Grs stand for almadine (Fe-Al) and grossular (Ca-Al) garnet, respectively (Reproduced with permission from Dai and Karato, Phys. Earth Planet. Inter.; published by Elsevier, 2009 [38]).

Dai and Karato [38] performed EC measurements in a series of dry and water-bearing pyrope-rich garnet single crystals (~Py_73_-Alm_14_-Grs_13_) at 873–1473 K, 4–16 GPa and a broad range of water content (<10 to 7000 ppm). The influence of the pressure on the EC of anhydrous and hydrous pyrope-rich garnet single crystals is shown in Figure 9 in detail. Under water-free conditions, the EC of pyrope-rich garnet was found to decrease slightly with increasing pressure while, the activation energy remains constant, with a relatively high value (Δ*E* = 128 kJ/mol). The effect of temperature is quite significant on the measured temperature range, causing the EC to increase by 3 orders of magnitude at any measured pressure. The hopping of small polaron was suggested as the main conduction mechanism. Under water-bearing conditions (160 ppm), the EC of garnet single crystal exhibits a weaker variation with pressure as compared to the dry sample, and the corresponding activation energy is much lower (70 kJ/mol). In this case, it has been proposed that the free proton is the charge carrier of the dominant conduction mechanism.

In subsequent studies, Dai et al. [39,40] measured the EC of a series of anhydrous and hydrous pyrope- and almandine-rich garnet single crystals with different chemical compositions (Py_20_Alm_76_Grs_4_, Py_30_Alm_67_Grs_3_, Py_56_Alm_43_Grs_1_, Py_73_Alm_14_Grs_13_ and Alm_82_Py_15_Grs_3_) under conditions of 1.0–4.0 GPa, 873–1273 K and five different solid buffers to control the oxygen fugacity (Fe_3_O_4_–Fe_2_O_3_, Ni–NiO, Fe–Fe_3_O_4_, Fe–FeO and Mo–MoO_2_).

The dependence relations of the EC of dry pyrope-rich, water-bearing pyrope-rich garnet (its water content: 465 ppm) and almandine-rich garnet single crystals on the oxygen fugacity were determined respectively as [39,40]:

(13) $$\log_{10}\sigma =\left(2.23\pm 0.063\right)+\left(0.061\pm 0.002\right)\times \log_{10}f_{O_{2}}+\frac{\left(-6092\pm 94\right)}{T}$$

(14) $$\log_{10}\sigma =\left(2.27\pm 0.032\right)+\left(-0.071\pm 0.001\right)\times \log_{10}f_{O_{2}}+\frac{\left(-6475\pm 48\right)}{T}$$

(15) $$\log_{10}\sigma =\left(2.67\pm 0.05\right)+\left(0.054\pm 0.003\right)\times \log_{10}f_{O_{2}}+\frac{\left(-5446\pm 68\right)}{T}$$

Similar to the previously-mentioned EC of olivine, the obtained values of *q*-exponent for garnet single crystal has be used to extrapolate different conduction mechanisms of mantle silicate minerals, either the hopping of small polaron ($Fe_{\mathrm{Mg}}^{•}$) or the hydrogen-related defects (*H^•^*, $H_{M}^{\prime }$ and ${\left(2H\right)}_{M}^{x}$). More recently, Liu et al. [107] investigated the EC of hydrous iron-rich garnet (40 and 100 ppm) in order to explain the high conductivity anomaly caused by the enrichment of eclogite region in the upper mantle.

**Figure 10:**
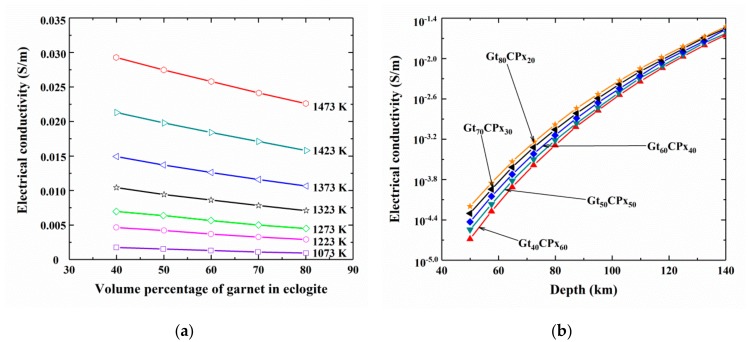
(**a**) The calculated electrical conductivity of eclogite as a function of the volume percentage of garnet, based on the obtained conductivity results of pyrope-rich garnet at conditions of 2.0 GPa, 1073–1473 K and the Ni–NiO oxygen buffer (modified from Dai et al. [39]). (**b**) The EC of eclogite at different volume percentages of the constituent minerals, i.e., garnet (Gt) and clinopyroxene (CPx) at depths of 40–140 km, at conditions of 2.0 GPa, 873–1473 K and the Ni–NiO solid oxygen buffer (Reproduced with permission from Dai et al., Contrib. Miner. Petrol.; published by Springer Nature, 2012 [39]).

Dai et al. [39], based on the obtained conductivity results of anhydrous pyrope-rich garnet (Py_73_Alm_14_Grs_13_) at 2.0 GPa, 873–1473 K and the Ni–NiO oxygen buffer combined with the effective medium theory, calculated the EC of eclogite as a function of the volume percentage of garnet (refer to Figure 10a). It is worth mentioning that eclogite is an important metamorphic rock as it is formed only under high-*P* conditions in the mantle. It is obvious that the EC of eclogite decreases almost linearly with increasing garnet content whereas, this tendency becomes weaker with decreasing temperature. Furthermore, the laboratory-based electrical conductivity-depth profile on eclogite with different volume ratios of garnet to clinopyroxene at the depths of 40–150 km was established by Dai et al. [39] at conditions of 2.0 GPa, 873–1473 K and the Ni–NiO solid oxygen buffer. Their results are shown in Figure 10b. Electrical conductivity of eclogite increases smoothly with depth, but at a slower rate as the depth increases. Furthermore, the EC of eclogite also increases with increasing the percentage volume of clinopyroxene, in agreement with reported results [105].

### 4.4. Electrical Conductivity of Wadsleyite and Ringwoodite

It is well known that olivine ((Mg,Fe)_2_SiO_4_) is the principal mineralogical phase in the shallow upper mantle region of the deep Earth interior. In the depth range of 410 to 660 km of the mantle transition zone, olivine is transformed to its high-pressure polymorphs of nominally anhydrous minerals: wadsleyite (~410 km depth) and ringwoodite (~660 km depth). High-pressure experiments and theoretical calculations have confirmed the high water storage capacity and solubility of wadsleyite and ringwoodite in the shallow mantle transition zone at the depth range of 410–660 km. A striking example of this huge water reservoir, is the discovery of hydrous ringwoodite, with high water content (1.4–1.5 × 10^4^ ppm as estimated by FTIR measurements), in the ultra-deep diamond inclusion below the lithospheric mantle in the region of the Juína district of Mato Grosso, Brazil [108].

Electrical conductivities of wadsleyite and ringwoodite were firstly measured by Xu et al. [41] under conditions of 15–20 GPa and at 1073–1473 K. They found that the phase transition from olivine to wadsleyite and ringwoodite can result in the EC jump by about a factor of 100 in the mantle transition zone. Subsequently, Huang et al. [11] measured the EC of hydrous wadsleyite and ringwoodite at conditions of pressure of 14–16 GPa, temperature of 773–1273 K, water content range from 100–10000 ppm and Mo–MoO_2_ oxygen buffer. At a given temperature and pressure, the EC of the sample increases with increasing water content. Based on the fitted Arrhenius relation between EC of hydrous sample and temperature, Huang et al. extrapolated the water content of ~1000–2000 ppm in the typical transition zone region, which corresponds to the EC values of ~10^−1^–5 × 10^−1^ S/m, within the temperature ranges of 1825–1900 K of the transition zone [11].

Karato and Dai [109] carried out EC measurements in dry and hydrous wadsleyite samples under conditions of 15 GPa and 873–1473 K. They found that the EC of dry and water-bearing olivine and wadsleyite is very close to each other, and the previously measured high electrical conductivities of wadsleyite and ringwoodite reported by Xu et al. [41] were assigned to the relatively higher concentration of structural water in their measured samples. They also stated that the observed conductivity jump at the depth of 410 km is possibly caused by the jump of the water content in the transition zone. Furthermore, Dai and Karato [3] performed EC measurements in synthetic polycrystalline wadsleyite using the Kawai-1000t multi-anvil pressure and an EIS analyzer. Their experiments were carried out at conditions of pressure of 15 GPa, temperature range of 873–1673 K, three solid oxygen buffers (e.g., Ni–NiO, Mo–MoO_2_ and Re–ReO_2_) and frequency range of 10^−2^–10^6^ Hz. They found that under really dry conditions, the wadsleyite aggregates with a low measured EC has a relatively high activation enthalpy of 147 kJ/mol. On the contrary, the hydrous wadsleyite samples with different water content have lower activation enthalpies (86–91 kJ/mol). The last finding implied that the electrical conductivities of dry and water-rich wadsleyite are related to different conduction processes, either small polaron or proton, and the total EC results from the following relation:

(16) $$\sigma =\sigma_{polaron}+\sigma_{proton}$$

For each conduction mechanism, the fitted equation of EC of the wadsleyite aggregates has been described as:

(17) $$\sigma_{i}=A\cdot C_{W}^{r}\cdot f_{O_{2}}^{q}\cdot \exp\left(-\Delta H/RT\right)$$

where, the quantities *C_W_*, $f_{O_{2}}$ and Δ*H* have the same meaning as in Equation (9) and the parameters *A*, *r*, *q* and *R* stand for constants [3].

**Figure 11:**
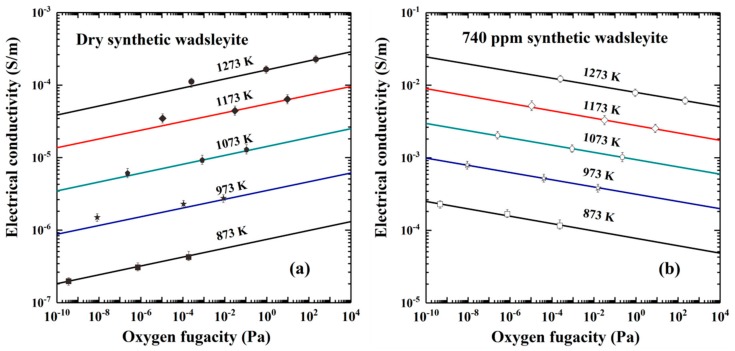
The influence of oxygen fugacity on the EC of (**a**) dry and (**b**) water-rich wadsleyite aggregates (740 ppm) under conditions of 15 GPa and 873–1273 K. Oxygen fugacity is controlled by three solid buffers, i.e., Mo–MoO_2_, Ni–NiO and Re–ReO_2_ (Reproduced with permission from Dai and Karato, Earth Planet. Sci. Lett.; published by Elsevier, 2009 [3]).

Figure 11 shows the EC of dry and water-rich wadsleyite aggregates (740 ppm) as a function of oxygen fugacity, under conditions of 15 GPa, 873–1273 K and three solid buffers (e.g., Mo–MoO_2_, Ni–NiO and Re–ReO_2_) [3]. At a given temperature and pressure, the EC of wadsleyite aggregates will be enhanced with increasing the water content (*C_W_*), which is expressed as $\sigma \propto C_{W}^{r}$ with the *r* value equal to ~0.72. Under anhydrous conditions, the EC of sample increases with increasing oxygen fugacity from the Mo–MoO_2_ to Re–ReO_2_ solid buffer. The dependence of EC on the oxygen fugacity, expressed by the *q*-exponent (0.050) can provide a robust evidence of an iron-related defect, such as small polaron of $Fe_{\mathrm{Mg}}^{•}$. However, the opposite tendency of EC along with oxygen fugacity is observed in hydrous wadsleyite, and the obtained negative *q*-exponent value (−0.058) revealed that some hydrogen-related defects can play a vital role in the electrical transport process in the transition zone. However, in both dry and hydrous samples, EC increases with increasing temperature from 873 to 1273 K. In comprehensive consideration of magnetotelluric sounding conductivity data (10^−2^–10^−1^ S/m) and mineralogical composition (60% of wadsleyite and 40% of majorite) of transition zone, the ~1000–3000 ppm of water is indispensable to account for the observed high conductivity anomaly in the Pacific region.

## 5. Some Remarks on the Evaluation of the Electrical Conductivity of Mantle Minerals

An important issue that has to be considered when interpreting the conductivity data is the frequency range of the applied ac-voltage that the measurements are carried out. Electrical impedance spectroscopy makes feasible to distinguish different conduction mechanisms in the overall electrical response of a measured mineral under HP-HT conditions, if the recorded spectrum ranges from a few mHz to several MHz, as it has been already mentioned in Section 2. However, misleading results can be obtained if the conductivity of the mineral under investigation is measured only at a single (low) frequency. This could be the case of hydrous minerals with different water content, where the interaction of water in the form of proton charges with the electrodes is significant, resulting in the formation of the electrical double layer (EDL) that manifests as a tail in the low frequency range of the impedance spectrum. For example, reported values of EC of wadsleyite by Manthilake et al. [13] and majorite garnet by Yoshino et al. [92,109] are lower than that measured by Dai and Karato [3,38] and Karato and Dai [109]. This underestimation of conductivity values in these cases is clearly justified by the higher electrical impedance measured at a low frequency value (0.01 Hz and 0.1 Hz), which actually appears increased due to the contribution of the electrodes polarization [109].

It is worth mentioning that the correct estimation of EC of minerals should be based on the proper selection of the equivalent circuit that fits satisfactory to the experimental data, which is obviously more complex than a simple R-C circuit in parallel that has been used in many cases in the past. Thus, the use of EIS is inevitable for the correct calculation of conductivity in hydrated minerals in polycrystalline form measured at high *T*, where ionic diffusion plays a significant role due to the charge accumulation in electrodes.

**Figure 12:**
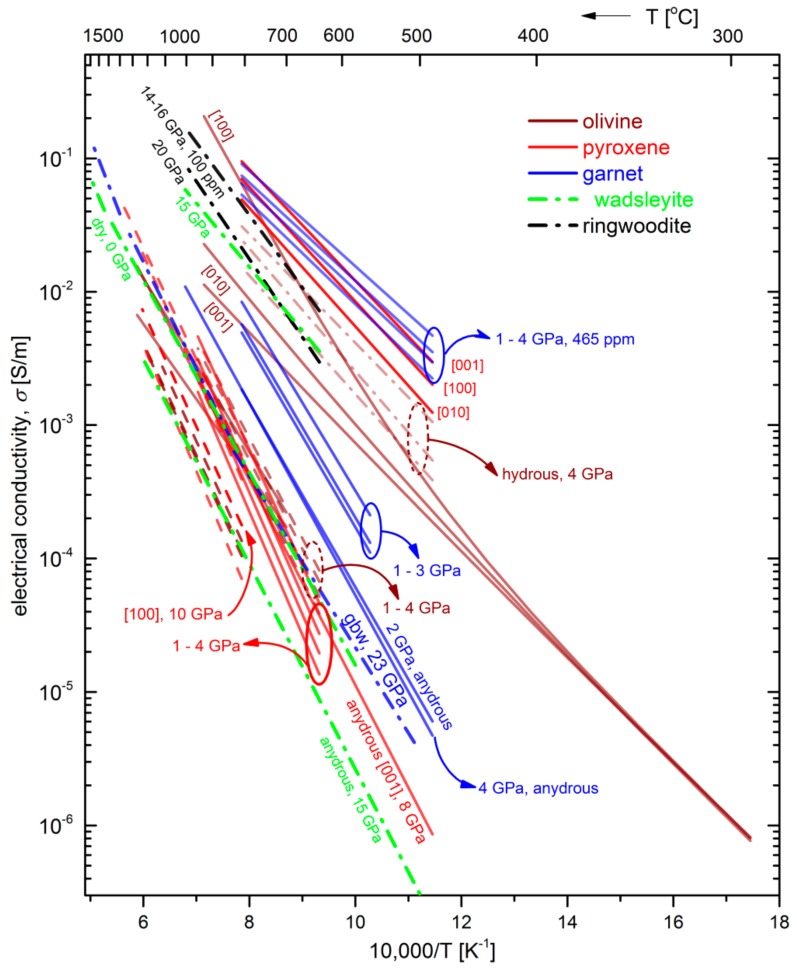
Arrhenius plot of EC of the major minerals in the upper mantle and the transition zone of Earth. The calculated parameters of the fitting lines are summarized in Table 1. The fitting lines are limited to the measured temperature and pressure ranges, without any extrapolations. Lines of the same color correspond to a certain mineral. Not all of the data in Table 1 are depicted in this figure, because of their large overlap. Solid lines correspond to single crystals and dashed lines to polycrystalline samples.

Based on the previous considerations about the necessity of EIS measurements for the proper estimation of EC of minerals, Figure 12 shows the Arrhenius plots of the EC of the major minerals discussed in the present work, derived exclusively from EIS measurements. The calculated parameters of the fitting data to the Arrhenius law according to Equation (5) are summarized in Table 1. We observe that, in almost all cases the Arrhenius behavior describes sufficiently the temperature dependence of the EC of the major mantle minerals. Furthermore, EC varies more than 5 orders of magnitude (10^−6^ S/m to 10^−1^ S/m), with the effect of temperature being stronger than that of pressure. According to the data presented in Table 1, the estimated activation enthalpy, Δ*H* of the dry minerals is higher than that of the hydrous minerals. In particular, for all the hydrous minerals, Δ*H* is less than 89 kJ/mol with the exception of polycrystalline ringwoodite, where Δ*H* reaches the value of 104 kJ/mol. On the contrary, in dry minerals, Δ*H* ranges between 108–180 kJ/mol. It is also remarkable that the anisotropic behavior of EC of olivine single crystals observed above moderate temperatures (T > 750 K) is more pronounced in the case of hydrous sample, while for the dry olivine sample, the degree of anisotropy is quite similar to that of the hydrous orthopyroxene [37]. In general, it is evident the necessity to perform measurements over a wider temperature range, especially at high temperatures (T > 1400 K) where ionic conductivity should be dominant.

In the cases where the pressure-dependence of EC has been measured, the important defect parameter of activation volume, Δ*V* has been determined, as it is shown in Table 1. The activation volume, defined as the pressure derivative of activation Gibbs free energy, $\Delta V=-{\left[\partial g_{act}/\partial P\right]}_{T}$, is related to the EC via the relation $\Delta V\approx -k_{B}T{\left[\partial \ln\sigma /\partial P\right]}_{T}$ and thus, a negative value is assigned to a process where $g_{act}$ (or conductivity) increases, as pressure increases. It is remarkable that, according to the reported values of ΔV in Table 1, a negative sign is observed in most cases of hydrous minerals, such as olivine and garnet [34,38,39] while, dry samples exhibit positive values of a few cm^3^/mol [38,40,41,64].

In ferromagnesian minerals, EC is mainly due to the diffusion of Mg^2+^ and Fe^2+^ cations, the charge transfer between Fe^3+^ and Fe^2+^ (or small polaron transport via the hopping mechanism) and the diffusion of H-related species, as it has been pointed out in Section 2.2 [54].

In the first case, diffusion takes place via the vacancy mechanism ($V_{M}^{\prime \prime }$) and the activation volume is the sum of the formation and migration volume of the vacancy defect, i.e., $\Delta V=V_{F}+V_{M}$. The formation volume *V_F_* of a mono-vacancy should be smaller than the atomic volume, due to the induced inward relaxation of the lattice while, the migration volume is usually much lower than the atomic volume. Thus, this conduction mechanism should be related to positive values of the activation volume, of a few cm^3^/mol. In the case of hopping of small polarons in minerals, reported conductivity measurements by Yoshino et al. indicate negative values of the activation volume [110]. As no formation of vacancies or interstitials is involved, negative values of ΔV should be related to an inward relaxation of the lattice during the charge transfer. Regarding the diffusion of H-related defects such as protons, the interstitial mechanism should dominate due to the small size of H, and thus, ΔV should only include the migration volume of the interstitial H-related defect, as no formation of defects is required. Taking into account that interstitial diffusion is generally weakly pressure-dependent, we expect that the values of ΔV for H conduction should be low and negative, if an inward relaxation takes place during the migration of H species from the equilibrium position to the saddle-point position. The case of negative activation volumes has been also reported in conductivity measurements of limestone samples and has been attributed to the enhanced proton conduction due to the pressure-induced dissociation of water located at the grain boundaries [111]. Papathanassiou et al. and Sakellis et al. [112,113,114,115] have also reported negative activation volumes in hydrated rocks such as leukolite, limestone, granodiorite and amphibolites. They concluded that water enhances the value of the negative Δ*V* and also lowers significantly the energetic threshold of the activated process.

As previously pointed out, the conduction mechanisms in hydrated minerals such as hydrogen-bearing olivine and its high-pressure polymorphs involve the diffusion of hydrogen-related defects and the diffusion of ions (Mg^2+^, Fe^2+^) at low and high temperatures, respectively. Thus, in a given temperature range dominated by a single conduction mechanism, electrical conductivity can be derived from the Nernst-Einstein relation, if the self-diffusion coefficients of an individual species are known in this temperature range. As a recent example, we can mention the work by Novella et al. [22] who studied the anisotropy of H self-diffusion in olivine single crystals at conditions of the upper mantle (2 GPa and 1023–1173 K) and calculated the H-enhanced EC of anisotropic and isotropic hydrous olivine by applying the Nernst-Einstein equation.

Opposed to the previous study, Karato [95,116] has proposed a theory to explain (a) the experimentally observed discrepancy of the activation enthalpies of electrical conductivity and diffusion in H-bearing olivine, and (b) the transition from isotropic behavior of olivine electrical conductivity observed at low-*T* to anisotropic one at high-*T*. According to this hybrid model, isotope diffusion coefficient is given by the harmonic mean of the different forms of hydrogen (free proton, one or two protons at M-site, etc.).

Regardless of the use of the harmonic mean to relate isotopic diffusion coefficients with the individual diffusion coefficients of the different forms of H, or the arithmetic mean of the diffusion coefficients of different defect species that contribute to the EC in minerals, the electrical conductivity-diffusion correlation could be treated on a thermodynamic basis, according to the so-called *cB*Ω thermodynamic model. This model proposed by Varotsos and Alexopoulos [117] describes effectively the defect processes in materials, by relating the self- or hetero-diffusion coefficients of a single mechanism with the elastic and expansion properties of the host material. Specifically, the activation Gibbs free energy, $g_{act}$ related to the formation and migration of a point defect is a function of the bulk thermo-elastic properties, i.e., $g_{act}=cB\Omega$, where *B* denotes the isothermal bulk modulus, Ω is the mean atomic volume and *c* is a constant that depends on the diffusion mechanism. Thus, the Arrhenius behavior of the diffusion coefficients is expressed as, $D\left(T,P\right)=D_{o}\exp\left(-cB\Omega /k_{B}Τ\right)$ and the point defect thermodynamic parameters, such as activation enthalpy, ΔH and activation volume, ΔV are given by the following relations [117]:

(18) $$\Delta H=c\Omega \left\{B-T\beta Β-Τ{\left.\frac{\partial Β}{\partial T}\right]}_{P}\right\},\;\;\Delta V=c\Omega \left\{{\left.\frac{\partial Β}{\partial P}\right]}_{T}-1\right\}$$

where *β* denotes the volume thermal expansion coefficient of the material which depends on temperature and pressure.

The significance of the model lies in its applicability to the case of ionic conduction in minerals observed at high-*T* or proton conduction at lower *T*, where the self-diffusion coefficients, and consequently the electrical conductivity calculated through the Nernst-Einstein relation, can be estimated over a broad temperature and pressure range, if the thermo-elastic properties of the mineral under investigation (i.e., *B*, ${\left.\partial Β/\partial T\right]}_{P},\;{\left.\partial Β/\partial P\right]}_{T}$ and *β*) are known in these *P-T* conditions. In addition, the important point defect parameter of activation volume can be calculated as a function of *T* and *P*, giving further insights to the investigation of the related conduction mechanism, as it has been pointed out previously [118,119,120].

The *cBΩ* model has been successfully implemented in diverse types of materials, including solid solutions and minerals [121,122,123,124]. Some recent examples of geophysical interest include the diffusion of He in olivine studied in the framework of the *cBΩ* model by Vallianatos and Saltas [53] and the work by Zhang and his coworkers who have studied self- and hetero-diffusion in (Mg,Fe)_2_SiO_4_ polymorphs, as well as hetero-diffusion (H, Na, K) in plagioclase feldspar [122,123,124]. In the latter case [124], the *cBΩ* model underestimates the electrical conductivity but gives some constraints to the reported experimental results.

## 6. Conclusions

Electrical conductivity of minerals (olivine, orthopyroxene, clinopyroxene, garnet, wadsleyite, ringwoodite, etc.) in the upper mantle and mantle transition zone are very sensitive to several factors including temperature, pressure, water content, crystallographic orientation and iron content. The proper determination of EC of minerals in a broad temperature and pressure range requires the utilization of complex electrical impedance spectroscopy measurements. The iron-related hopping of small polaron and the hydrogen-related defects are possibly the two dominant conduction mechanisms in anhydrous and hydrous Fe-bearing silicate minerals within the depth range of mantle. The trace structural water of mantle minerals plays a crucial role in explaining the high conductivity anomaly and the water distribution in the deep mantle. By combining self-diffusion measurements in minerals over a narrow temperature range with their thermo-mechanical properties in an extended range of *P-T*, the EC can be estimated by the Nernst-Einstein equation, applying a well-established thermodynamic model. This can shed more light on the underlying conduction mechanisms at high-*T* and high-*P* conditions, thus extending the use of conductivity profiles to geophysical models.

## Figures and Tables

**Table 1:** Fitting parameters of the Arrhenius behavior of electrical conductivity ($\sigma =\sigma_{ο}\cdot \exp\left(-\Delta H/RT\right)$) of the major minerals in the upper mantle and transition zone, at different *P-T* conditions, measured exclusively by the impedance spectroscopy technique. The pre-exponential term *σ*_ο_, may include the contribution of water content *C*_w_ and oxygen fugacity, where applicable.

Mineral	-	*P* (GPa)	*T* (K)	*C*_W_ (ppm H/Si)	log*σ_ο_* (S/m)	Δ*H* (kJ/mol)	Δ*V* (cm^3^/mol)	Ref.
olivine	single crystal ^a^ [100]	4	573–1373	1380 (SIMS cal.)	0.74 ± 0.09, 4.52 ± 0.20	75 ± 2, 140 ± 6	-	[35]
	[010]				0.51 ± 0.04, 1.99 ± 0.16	73 ± 3, 101 ± 3	-	
	[001]				0.30 ± 0.10, 1.08 ± 0.13	71 ± 2, 87 ± 5	-	
	hydrous polycrystalline, Re-ReO_2_ buffer	4	873–1273	3675 (3628) ^b^	1.67 ± 0.08	85 ± 5	-	[33]
	Ni-NiO buffer			2853 (2737)	2.00 ± 0.13	88 ± 3	-	
	Mo-MoO_2_ buffer			4406 (4394)	1.69 ± 0.10	78 ± 4	-	
	hydrous polycrystalline	4	873–1273	2335 (2317)	2.67	88.6 ± 4	−0.86 ± 0.05	[34]
		7		2326 (2296)	2.63	86.0 ± 4		
		10		2329 (2434)	2.58	83.4 ± 4		
	dry polycrystalline, Mo-MoO_2_ buffer	1	1073–1423	not measured	2.91 ± 0.12	143.8 ± 2.7	0.25 ± 0.05	[64]
		2			2.93 ± 0.17	145.9 ± 2.0		
		3			2.93 ± 0.10	148.2 ± 2.3		
		4			2.96 ± 0.13	151.4 ± 3.1		
	single crystal San Carlos (Fo_90_), [100]	8	1123–1709	nominally dry	2.52	140.9	-	[89]
	[010]				1.14	108.1	-	
	[001]				2.00	124.5	-	
	polycrystalline Mg_1.8_Fe_0.2_SiO_4_	4	1273–1573	not measured	2.98 ± 0.17	166.9 ± 4.8	0.68 ± 0.14	[41]
		7	1273–1673		2.63 ± 0.19	160.2 ± 4.8		
		10	1273–1673		2.71 ± 0.18	163.1 ± 4.8		
pyroxenes	polycrystalline orthopyroxene	5	1273–1673	not measured	3.72 ± 0.06	173.7 ± 1.9	-	[44]
	polycrystalline clinopyroxene	13	1273–1673		3.25 ± 0.07	180.4 ± 1.9	-	
	polycrystalline ilmenite + garnet	21	1473–1773		3.35 ± 0.10	160.2 ± 2.9	-	
	anhydrous orthopyroxene [001]	8	873–1473	<8	2.73 ± 1.18	147 ± 7	-	[37]
	hydrous orthopyroxene [001]		873–1273	4660 (4540)	2.26 ± 1.00	80 ± 2	-	
	[100]		873–1273	4690 (4600)	2.21 ± 1.11	82 ± 3	-	
	[010]		873–1273	4700 (4640)	2.18 ± 0.95	85 ± 2	-	
	single crystal, [100]	10	1273–1673	not measured	3.02 ± 0.22	165.0 ± 5.8	-	[41]
	orthopyroxene single crystal	1	1073–1423	not measured	3.79	166.9	0.03 ± 0.01	[90]
		2			3.80	171.8		
		3			3.79	174.6		
		4			3.73	176.6		
garnets	pyrope-rich single crystal, anhydrous	4–16	873–1473	<10	2.93–2.48	138–168	2.50 ± 0.48	[38]
	hydrous	4, 8	873–1273	7000	1950$\cdot C_{w}^{0.63}$	67.7, 65.4	−0.57 ± 0.05	
	anhydrous Py_73_Alm_14_Grs_13_ single crystal	2	873–1273	<1	2.69	132.2	-	[39]
	hydrous Py_73_Alm_14_Grs_13_ single crystal	1	873–1273	465	1.735	73.3	−1.4 ± 0.15	[39]
		2			1.742	71.4		
		3			1.759	70.4		
		4			1.769	68.5		
	almandine-rich garnet single crystal	1	973–1273	<3 (6)	3.11	126.4	2.01 ± 0.57	[40]
		2			3.06	129.3		
		3			3.04	130.3		
	majorite garnet	23	900–1300	grainboundarywater	1.73	122.5	-	[91]
			1300–1750		3.03	153.4		
			1800–2000		4.24	194.9		
wadsleyite	polycrystalline (5–10 μm)	14–16	773–1273	10^2^–10^5^	2.6 + 0.66log*C_W_*	88 ± 3	-	[11]
	anhydrous polycrystalline (9 μm)	15	873–1673	<9	2.1 ± 0.1	147 ± 3	-	[3]
	hydrous polycrystalline (3–7 μm)		873–1273	360–32000	2.5 + 0.72log*C*_W_	88 ± 10	-	
	polycrystalline (20 μm)	ambient	1000–1850	<2	2.41	138.0	-	[92]
			1900–2000		3.57	180.4	-	
	polycrystalline	15	1073–1473	not measured	2.04 ± 0.15	91.7 ± 3.9	-	[41]
ringwoodite	polycrystalline (5–10 μm)	14–16	773–1273	10^2^–10^5^	3.6 + 0.69log*C*_W_	104 ± 2	-	[11]
	polycrystalline	20	1073–1473	not measured	2.92 ± 0.04	111.9 ± 1.0	-	[41]

^a^ Two conduction mechanisms of Arrhenius behavior have been considered ^b^ Numbers in parentheses denote water content after the experiment.

## Abbreviations

CPE	Constant phase element
COMPRES	Consortium for Material Properties Research in Earth Sciences
EIS	Electrical impedance spectroscopy
EC	Electrical conductivity
FTIR	Fast Fourier infra-red
HP	High pressure
HT	High temperature
HTHPSEI	High-temperature and High-pressure Study of the Earth’s Interior
NAM	Nominally anhydrous mineral
WC	Tungsten carbide

## Nomenclature

*σ* (S/m)	Complex electrical conductivity
*σ_o_* (S/m)	Pre-exponential factor
Δ*E* (kJ/mol)	Activation energy
Δ*H* (kJ/mol)	Activation enthalpy
Δ*V* (cm^3^/mol)	Activation volume
*C_w_* (ppm)	Water content (in H/10^6^Si)
$f_{O_{2}}$(Pa)	Oxygen fugacity
*Z** (Ω)	Complex impedance
*B*	Isothermal bulk modulus
*β*	Volume thermal expansion coefficient
